# Alpha and Beta Oscillations Differentially Support Word Production in a Rule-Switching Task

**DOI:** 10.1523/ENEURO.0312-23.2024

**Published:** 2024-03-29

**Authors:** Ioanna Zioga, Ying Joey Zhou, Hugo Weissbart, Andrea E. Martin, Saskia Haegens

**Affiliations:** ^1^Donders Centre for Cognitive Neuroimaging, Donders Institute for Brain, Cognition and Behaviour, Radboud University, Nijmegen 6525 EN, The Netherlands; ^2^Max Planck Institute for Psycholinguistics, Nijmegen 6525 XD, The Netherlands; ^3^Department of Psychiatry, Oxford Centre for Human Brain Activity, Oxford, United Kingdom; ^4^Department of Psychiatry, Columbia University, New York, New York 10032; ^5^Division of Systems Neuroscience, New York State Psychiatric Institute, New York, New York 10032

**Keywords:** alpha oscillations, beta oscillations, cue, exemplar, feature, MEG

## Abstract

Research into the role of brain oscillations in basic perceptual and cognitive functions has suggested that the alpha rhythm reflects functional inhibition while the beta rhythm reflects neural ensemble (re)activation. However, little is known regarding the generalization of these proposed fundamental operations to linguistic processes, such as speech comprehension and production. Here, we recorded magnetoencephalography in participants performing a novel rule-switching paradigm. Specifically, Dutch native speakers had to produce an alternative exemplar from the same category or a feature of a given target word embedded in spoken sentences (e.g., for the word “tuna”, an exemplar from the same category—“seafood”—would be “shrimp”, and a feature would be “pink”). A cue indicated the task rule—exemplar or feature—either before (pre-cue) or after (retro-cue) listening to the sentence. Alpha power during the working memory delay was lower for retro-cue compared with that for pre-cue in the left hemispheric language-related regions. Critically, alpha power negatively correlated with reaction times, suggestive of alpha facilitating task performance by regulating inhibition in regions linked to lexical retrieval. Furthermore, we observed a different spatiotemporal pattern of beta activity for exemplars versus features in the right temporoparietal regions, in line with the proposed role of beta in recruiting neural networks for the encoding of distinct categories. Overall, our study provides evidence for the generalizability of the role of alpha and beta oscillations from perceptual to more “complex, linguistic processes” and offers a novel task to investigate links between rule-switching, working memory, and word production.

## Significance Statement

It remains unclear whether the proposed functional role of alpha and beta oscillations in perceptual function is generalizable to higher-level cognitive processes. We constructed a novel rule-switching paradigm involving speech comprehension and word production. We found that alpha power is modulated by cognitive load and is linked to task performance, potentially by regulating inhibition in brain regions linked to lexical retrieval. Additionally, the spatiotemporal pattern of beta activity differed between two distinct task rules, in line with the proposed role of beta in encoding of distinct categories and recruitment of respective neural networks. We offer experimental findings that support the view of a domain-general role of oscillations across the hierarchy of cognitive functions, from low-level sensory operations to high-level processes.

## Introduction

One prominent view on brain oscillations is that they provide the scaffolding that supports various cognitive functions in a dynamic and task-dependent manner (Bishop, 1932; Rassi et al., 2023). Previous research investigated the role of brain oscillations in basic operations, such as working memory and rule-switching (Buzsáki and Draguhn, 2004; Akam and Kullmann, 2010; Miller et al., 2018). However, little is known regarding the generalization of these operations as identified in lower-level (perceptual) tasks to more complex functions. Here, we designed a novel rule-switching task involving speech comprehension and word production to test the role of alpha and beta oscillations in a linguistic setting.

Alpha oscillations (8–14 Hz) are proposed to orchestrate processing by inhibiting task-irrelevant regions and facilitating activation of task-relevant regions (Klimesch et al., 2007; Jensen and Mazaheri, 2010). Alpha power negatively correlates with neural excitability (Haegens et al., 2022; Iemi et al., 2022). Previous studies found alpha power modulations during memory retention (Bonnefond and Jensen, 2013; van Ede et al., 2017; de Vries et al., 2020; Rodriguez-Larios et al., 2022) and associations with behavioral performance (Haegens et al., 2010, 2012; ElShafei et al., 2022).

Beta oscillations (15–30 Hz) are linked to top-down processing via reactivation of representations by supporting neural ensemble formation (Engel and Fries, 2010; Spitzer and Haegens, 2017). Across primate studies, beta dynamics appear content specific, carrying information about task rules (Buschman et al., 2012), stimulus categories (Antzoulatos and Miller, 2016), scalar magnitudes (Spitzer and Blankenburg, 2011), and decision outcomes (Haegens et al., 2011b, 2017). Rassi et al. (2023) recently found that networks in the dorsolateral prefrontal cortex and presupplementary motor area synchronize at distinct beta-frequency rhythms and reflect the animal's categorical decision.

The oscillatory correlates of language processing have been widely investigated (Rommers et al., 2017; Meyer, 2018; Prystauka and Lewis, 2019; Brennan and Martin, 2020; Weissbart et al., 2020; Hustá et al., 2021; ten Oever and Martin, 2021; Bai et al., 2022; Coopmans et al., 2022; ten Oever et al., 2022; Slaats et al., 2023). Alpha/beta desynchronization has been interpreted as conceptual-lexical retrieval (Piai et al., 2014; Cao et al., 2022). Alpha/beta power was also modulated by syntactic features while listening to comprehensible but not incomprehensible stories (Zioga et al., 2023). Alpha modulations are associated with attention during word-level prediction (Stinkeste et al., 2023), while beta oscillations are thought to carry top-down predictive information during sentence processing (Bastiaansen et al., 2010; Lewis and Bastiaansen, 2015). However, the role of oscillations in language processing has been widely studied in isolation to their well-established role in low-level processes.

Our goal was to study alpha and beta dynamics incorporating working memory, language comprehension, and word production demands. Participants were required to generate an exemplar or a feature based on given target words embedded in spoken sentences. In each trial, a cue indicated the task rule (exemplar/feature) either before listening to the sentence (pre-cue) or after the sentence and a delay (retro-cue). The rationale for the exemplar versus feature production was twofold: (1) tapping into distinct cognitive processes (as exemplar generation requires conceptual retrieval within a category, whereas feature generation requires naming a property of an exemplar that can span over different categories) allowed us to test the role of beta in the recruitment of distinct networks; and (2) both are generative processes with open-ended responses. Finally, the use of sentences provides preactivation of related concepts, promoting the generation of a larger number of, and more original, ideas (Howard-Jones and Murray, 2003; Liu, 2016).

Previous intracranial work identified distinct frontotemporal networks for sentence reading, sensitive to sentence- and lexical-level meaning, respectively (Woolnough et al., 2023). Research on aphasic patients suggested that the left hemispheric frontotemporal language regions are selectively engaged in language by storing domain-specific knowledge representations, whereas nonlinguistic abilities do not rely on the language system (Fedorenko and Varley, 2016). Additionally, a visual retro-cueing study found decreased prefrontal and occipitoparietal alpha/beta power with increased cognitive load (ElShafei et al., 2022). Similarly, primate research revealed beta activity in prefrontal and motor areas involved in the reactivation of representations corresponding to distinct categorical decisions (Rassi et al., 2023). We hypothesized alpha power in left language-related regions to be modulated based on cognitive load, that is, higher alpha power for pre-cue, lower for retro-cue. We further hypothesized distinct task rules (exemplar/feature) to exhibit a differentiated spatiotemporal profile of beta band activity during the delay window, reflecting the recruitment of distinct networks, potentially spanning over nonlinguistic regions.

## Materials and Methods

### Participants

Thirty-three adults (23 female) aged between 19 and 39 years (M ± SD age of 25.4 ± 5.6 years) took part in the experiment. Prescreening required that participants were right-handed Dutch native speakers, without hearing problems, reading problems, or epilepsy. We encountered technical problems during six recordings, leaving us with 27 participants for the magnetoencephalography (MEG) analysis. Prior to the experiment, participants were provided with written and verbal information and gave written informed consent. They received monetary reimbursement after participation. The study falls under the general ethics approval (CMO 2014/288 “Imaging Human Cognition”) in accordance with the Declaration of Helsinki.

### Experimental task

**Figure 1.**
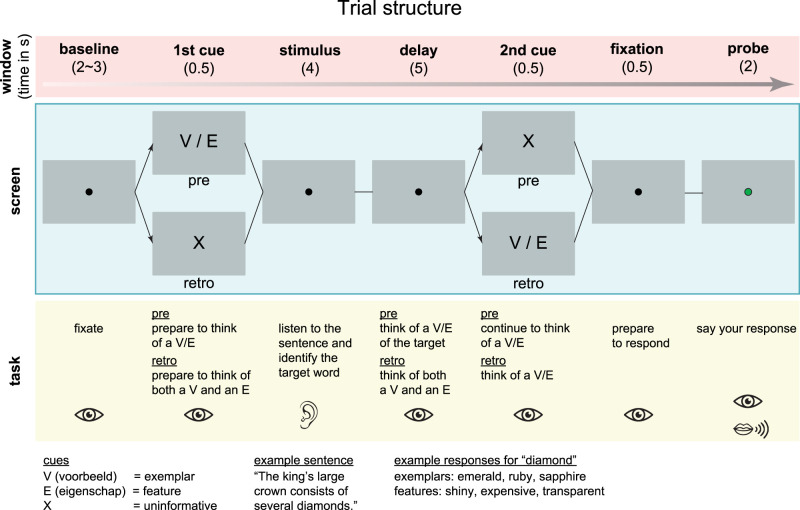
Trial structure. The experimental stimuli were in Dutch; for illustrative purposes, the English translation is shown here.

We designed a novel rule-switching task involving linguistic demands. Participants listened to sentences and performed a word production task based on cues (Fig. 1). In the pre-cue condition, after an intertrial interval of 2–3 s (duration drawn from a normal distribution), the first cue was presented on screen (0.5 s). The cue was either a “V” for exemplar (“voorbeeld” in Dutch) or an “E” for feature (“eigenschap”), informing participants of the task rule (exemplar or feature) at the beginning of the trial. Then, they listened to a 4 s sentence binaurally through earphones, in which they had to identify a target word. Participants were “informed” that the target word was either the penultimate or last word of a sentence and that it was always a noun. The sentences were designed such that only one of the final two words was a noun, to make finding the target word unambiguous. They were given a 5 s delay period to come up with an alternative exemplar from the same category (if the first cue was a V) or a feature of the given target word (if the first cue was an E). Then, they were presented with an uninformative second cue (an “X” for 0.5 s). Participants were prompted to verbalize their answer as soon as a centrally presented dot turned green. The retro-cue condition was exactly the same as the pre-cue condition, except that here the first cue (before sentence presentation) was uninformative, while the second cue (after sentence presentation) indicated the exemplar or feature task rule. Participants were instructed to come up with both an exemplar and a feature during the delay period and found out which of the two to verbalize at second cue presentation. Note that the uninformative cues, while not providing information regarding the task rule, were included to keep the two cueing conditions identical in terms of temporal structure. The experiment consisted of 300 trials, presented in 12 blocks of 25 trials each with a self-paced break between each block. Trial order and conditions were randomized within and across blocks.

### Sentence stimuli

The stimulus set was composed of 150 Dutch sentences (all materials will be made publicly available upon acceptance of the manuscript). Sentences were constructed by the authors in collaboration with a Dutch native speaker. The target word was an exemplar of a category (e.g., “diamonds” was an exemplar of the inferred category “precious stones”). To ensure that participants paid attention until the end of the sentence, in half of the sentences the target word came last, whereas in the other half the target word was the penultimate word of the sentence. Each sentence was presented twice, once syntactically intact (structured) and once with the words in shuffled order (scrambled). To ensure that the identification of the target word was equally possible in both sentence conditions, the order of the last two words (one of which was the target word) was identical between structured and scrambled conditions. (Note that the structured vs scrambled manipulation was outside of the scope of the current analysis.) Google Text-to-Speech was used to create synthesized speech from text with the following parameters: voice_name = “nl-NL-Wavenet-B”, voice_ssml_gender = texttospeech.SsmlVoiceGender.MALE, and sr_hertz = 44,100. Sentences had a duration of 4 s. To remove abrupt sound bursts, the first and last 5% of the 4 s sound clips were smoothed by a linear ramp (a cosine wave). The root-mean-square value of each sentence was normalized to −16 dB to control the stimulus intensity.

### Procedure

Participants were seated in the MEG system in a dimly lit room and were presented with instructions on screen about the task. They were informed that they would listen to sentences in Dutch and perform an exemplar versus feature word production task. Specifically, they would hear a sentence and would need to identify a target word, that is, a noun which would be either the last or penultimate word of the sentence. They were told that their task would be to come up with an alternative exemplar from the same category or a feature of the given target word. Exemplars were defined as concepts that belong to a category (e.g., “tuna” belongs to the category “fish,” other exemplars from the same category are cod, lobster, squid), while features were defined as attributes that describe a concept (e.g., “tuna”: edible, proteins, gills). Only single-word answers were accepted. Then, participants were informed about the cues based on which they would produce their answers: V for exemplar (“voorbeeld” in Dutch), E for feature (“eigenschap” in Dutch), and X for both exemplar and feature in the pre-cue or for uninformative in the retro-cue.

Before the actual experiment, participants completed a practice session in which they were familiarized with all aspects of the task. To practice correctly identifying the target word, they were presented with 10 sentences and were asked to identify and verbalize the target word in each. The correct target word was then presented on screen. Next, they were asked to perform the experimental task in a self-paced manner, by pressing a key on a response box when they were ready to move to the next screen. This part consisted of one trial in each of the four subconditions (pre-cue exemplar, pre-cue feature, retro-cue exemplar, and retro-cue feature). Finally, participants completed 12 trials identical to the actual task, three for each condition.

After the practice session, participants performed the experimental task consisting of 300 trials while their MEG was recorded. Sentence order was randomized across participants. The overall procedure lasted ∼1.5 h. The experiment was programmed in MATLAB (MathWorks) using Psychtoolbox (Brainard and Vision, 1997).

### Data acquisition

MEG data were recorded at a sampling rate of 1,200 Hz using a 275-channel axial gradiometer system (CTF MEG systems, VSM MedTech) located in a magnetically shielded room. Eight sensors were disabled due to permanent malfunction, leaving a total of 267 usable sensors. Three fiducial localization coils were placed at the participant's nasion and left and right ear canals to (1) allow for real-time monitoring (Stolk et al., 2013) of the participant's head position and adjustment in between experimental blocks if necessary and (2) provide anatomical landmarks for offline coregistration of the MEG data with T1-weighted MRI images for source reconstruction. After completion of the task, the coordinates of the three fiducial points as well as the participant's head shape were digitized using a Polhemus 3D tracking device (Polhemus). Individual structural MRI scans were acquired in a 3 T Siemens Magnetom Skyra MR scanner using earplugs with a drop of vitamin E at the participant's ear canals to facilitate subsequent alignment with the MEG data.

### Behavioral data analysis

#### Accuracy

A Dutch native speaker naive to the purpose of the experiment evaluated the validity of participants’ responses, to ensure that they performed the task appropriately, and to exclude from the analysis trials in which they did not successfully perform the task (i.e., gave no response, gave an invalid response, or gave a response that was the opposite task rule compared with the given cue). A 2 (cue: pre, retro) × 2 (task rule: exemplar, feature) repeated measures ANOVA was performed on participants’ accuracy quantified as percentage of correct responses.

#### Reaction times

Reaction times were marked based on the voice onset time of the verbal response relative to the onset of the probe. Only correct responses were analyzed. We conducted a 2 (cue: pre, retro) × 2 (task rule: exemplar, feature) repeated measures ANOVA on reaction times.

#### Response word frequency

Word frequency is a property of each individual word out of context. To estimate the word frequency of participants’ responses, the online database SUBTLEX-NL was used (Keuleers et al., 2010). For each response given by our participants, the number of instances of that response within the corpus used by SUBTLEX-NL was divided by the total number of instances of all words in the same corpus, and then the logarithm base 10 of that number was calculated (the higher the value, the higher the word frequency). A 2 (cue: pre, retro) × 2 (task rule: exemplar, feature) repeated measures ANOVA was conducted.

#### Response variability

As we wanted to test whether task rule had an impact on the variability of responses within our sample, we employed a measure of how variable the response to a particular target word was across participants. A 2 (cue: pre, retro) × 2 (task rule: exemplar, feature) repeated measures ANOVA was performed on intersubject variability, defined as the number of different responses that were provided for each target word. Response variability thus comprised a word-level metric, in contrast to the aforementioned measures, which were computed at the participant level.

#### Predicting reaction times from behavioral measures

To test which variables explain variability in reaction times, we constructed generalized linear mixed models (GLMMs) with participants as the random-effects predictor, while word frequency, cue, and task rule were candidate fixed effects (Table 1). Likelihood ratio tests (LRTs) were used to compare the goodness of fit of nested models to determine whether or not adding complexity to the models made them significantly more accurate.

**Table 1.** Model fit statistics and log likelihood comparisons between mixed-effects regression models predicting reaction times

Model *n*	Comparison	Predictors	df	*χ* ^2^	*p*
Behavioral GLMMs					
1	-	WF	4	−411.31	ΝΑ
2	2 vs 1	WF + cue	5	−211.88	<0.001
3	3 vs 1	WF + task	5	−396.16	0.001
4^a^	4 vs 2^a^	WF + cue*X*task^a^	7^a^	−185.58^a^	<0.001^a^
5	5 vs 4	WF*X*cue*X*task	10	−184.56	0.564
Neural GLMMs					
1	-	IAF	4	−485.49	ΝΑ
2	2 vs 1	IAF + cue	5	−260.17	<0.001
3	3 vs 1	IAF + task	5	−460.33	<0.001
4^a^	4 vs 2^a^	IAF + cue*X*task^a^	7^a^	−224.57^a^	<0.001^a^
5	5 vs 4	IAF*X*cue*X*task	10	−222.74	0.301

Model *n*: model number. Comparison: contrasted models. Predictors: predictors included in the model. LRT, log likelihood ratio test; WF, word frequency; Task, task rule; IAF, individual alpha peak frequency power at left frontal sensors; *χ*^2^, chi-squared test. *X* denotes main effect and interaction.

a Best model based on likelihood ratio tests.

### MEG sensor-level analysis

#### Preprocessing

Continuous MEG data were downsampled to 200 Hz. Data from sensors with consistently poor signal quality, as observed by visual inspection, were removed and interpolated based on neighboring sensors. Finally, independent component analysis (Jung et al., 2001) was performed to correct for eyeblinks and heartbeat artifacts. Custom MATLAB code and the FieldTrip toolbox (Oostenveld et al., 2011) were used for analysis of the MEG data.

#### Time-frequency representation (TFR)

To compare oscillatory brain activity between conditions (pre vs retro-cue, exemplar vs feature), we conducted a time-frequency analysis from −0.5 to 11.5 s time-locked to the presentation of the first cue (time 0). We used only trials in which participants gave a valid response. The MEG signal was convolved with a sliding Hanning taper (adaptive window length), and the TFR was calculated from 7 to 30 Hz in 50 logarithmically spaced steps, using 6-cycle wavelets, with a time step size of 100 ms. Finally, power values were converted to decibel by calculating the 10 times base 10 logarithm of the ratio of the TFR after time 0 and the average baseline power from −0.45 to −0.25 s.

#### Nonparametric cluster-based permutation test

We used a nonparametric cluster-based permutation approach to compare the TFRs between conditions (Maris and Oostenveld, 2007). The test range was from 7 to 30 Hz and from −0.5 to 11.5 s, for all sensors. Paired-sample *t* tests were computed for each sensor, frequency, and time point. Spectro-spatio-temporally adjacent datapoints whose *t* values exceeded an a priori threshold (uncorrected *p* value <0.05) and had the same sign (positive or negative) were combined into clusters, with the cluster-level statistic calculated as the sum of the *t* values of the cluster. Finally, the values of the cluster-level statistic were evaluated by calculating the probability that it would be observed under the assumption that the two compared conditions are not significantly different (*α* = 0.05; two-tailed). To obtain a null distribution to evaluate the statistic of the actual data, values were randomly assigned to the two conditions and the statistics re-computed 1,000 times (Monte-Carlo permutation).

#### Predicting reaction times from individual alpha peak frequency power

To investigate potential links between alpha power and behavioral performance, we constructed GLMMs predicting reaction times from individual alpha peak power (Table 1). To identify the individual alpha peak frequency (IAF) per participant, we computed the power spectra of the MEG data during the baseline period (−1 to 0 s) to avoid biasing the estimation of the IAF with task demands. Based on previous work (ElShafei et al., 2022), we then identified the top 20 sensors with the maximal power, calculated their average power spectrum, and detected the local maximum within the 8–13 Hz range. IAF was defined as the frequency bin with the highest power in the alpha band. Across participants, M ± SD IAF was 10.2 ± 1.3 Hz. Note that we only used the peak 20 sensors to identify the IAF, while we used all sensors for the GLMM analysis predicting reaction times from IAF power.

### MEG source reconstruction

For each participant, coregistration of the MRI with the CTF and Polhemus fiducials was performed. Individual MRIs were normalized in MNI space (International Consortium for Brain Mapping, Montreal Neurological Institute, Canada) and segmented. Realistic volume conduction models were created for each participant based on the single-shell model of their MRIs (Nolte, 2003). An 8 mm resolution resulted in 5,798 dipole positions. To localize the sources of our observed effects, we performed beamformer analysis at the frequency of interest using an adaptive spatial filtering technique (Dynamic Imaging of Coherent Sources, DICS; Gross et al., 2001), focusing on the time and frequency windows where the sensor-level effects peaked. For the pre-cue versus retro-cue contrast, we analyzed the following time windows: delay window (8.5–9.5 s), presentation of second cue (9.5–10 s), fixation (10–10.5 s), and probe/word production (10.5–11.5 s). For the exemplar versus feature contrast, we analyzed two 1 s nonoverlapping time windows of the delay period: 6–7 s and 7–8 s. Cross-spectral density matrices were computed using Fourier transform on data from both conditions combined. Based on the spectral effects identified in the sensor-level analysis, for pre versus retro-cue, alpha band was estimated at 10 ± 3 Hz and beta at 20 ± 5 Hz, while for exemplar versus feature alpha band was at 11 ± 1 Hz and beta at 17 ± 4 Hz. A common spatial filter was calculated for each participant, which was then applied to each condition separately to extract the source-level power estimates. The regularization parameter was set to 5%. Finally, the Brainnetome Atlas (Fan et al., 2016) was used to identify the regions (parcel labels) where the maximal effects were found based on the peak *t* values. This atlas divides each hemisphere into 123 parcels, based on both structural and functional connectivity.

### Code accessibility

The data and MATLAB code to reproduce our findings are freely available on the Donders Repository (https://data.donders.ru.nl) at https://doi.org/10.34973/geks-ge93.

## Results

### Cue and task rule modulate reaction times but not accuracy

**Figure 2.**
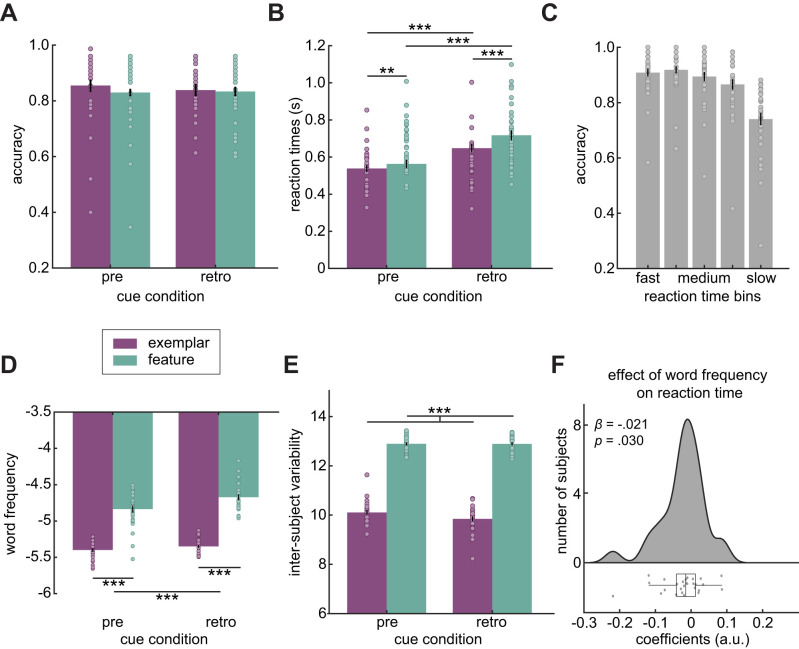
Behavioral performance. ***A***, Accuracy (rate of correct responses), separately for pre- versus retro-cue trials and for exemplars (purple) versus features (green). ***B***, Reaction times. ***C***, Speed–accuracy trade-off: rate of correct responses for fast to slow reaction time bins. ***D***, Logarithm base 10 of the word frequency of responses. ***E***, Intersubject variability (how many different responses were given over all participants). ***F***, Results from a generalized linear mixed-effects model predicting reaction times from word frequency with subject as random-effects predictor. For plotting purposes, the raincloud plot shows coefficients of a GLM per subject. ***p < 0.001, **p < 0.010.

To verify that participants completed the task as instructed and that there were a sufficient number of trials available for the MEG analysis, accuracy was computed as the rate of correct answers (as assessed by a Dutch native speaker). A 2 (cue: pre, retro) × 2 (task rule: exemplar, feature) repeated measures ANOVA on accuracy showed no effect or interaction between the variables (*p* > 0.1; Fig. 2*A*).

Reaction times were longer for the retro-cue than pre-cue condition (significant main effect of cue: *F*_(1,32)_ = 95.526; *p* < 0.001; partial *η*^2^ = 0.749) and longer for feature than exemplar production (main effect of task rule: *F*_(1,32)_ = 17.970; *p* < 0.001; partial *η*^2^ = 0.360). There was also a significant interaction between the variables (*F*_(1,32)_ = 5.018; *p* = 0.032; partial *η*^2^ = 0.136). Planned contrasts showed that reaction times were longer for retro-cue than pre-cue conditions for both exemplars (*t*_(32)_ = 8.502; *p* < 0.001; Cohen's *d* = 1.480) and features (*t*_(32)_ = 7.768; *p* < 0.001; Cohen's *d* = 1.352). Furthermore, reaction times were longer for features than exemplars for both pre-cue (*t*_(32)_ = 2.895; *p* = 0.007; Cohen's *d* = 0.504) and retro-cue conditions (*t*_(32)_ = 3.608; *p* = 0.001; Cohen's *d* = 0.628; Fig. 2*B*).

There was no speed–accuracy trade-off, that is, slower reaction times were associated with lower accuracy, confirmed by a significant linear regression (*R*^2^ = 0.221; *p* < 0.001; Fig. 2*C*).

### Cue and task rule modulate word frequency and variability of responses

To test whether word frequency of the responses was modulated by condition, we conducted a 2 (cue: pre, retro) × 2 (task rule: exemplar, feature) repeated measures ANOVA. Word frequency was lower for pre- compared with retro-cue trials (main effect of cue: *F*_(1,32)_ = −17.255; *p* < 0.001; partial *η*^2^ = 0.350) and for exemplars compared with features (main effect of task rule: *F*_(1,32)_ = 95.899; *p* < 0.001; partial *η*^2^ = 0.750). There was also a significant interaction between the variables (*F*_(1,32)_ = 4.466; *p* = 0.042; partial *η*^2^ = 0.122). Planned contrasts showed that pre-cue was associated with lower word frequency responses than retro-cue for features (*t*_(32)_ = −4.293; *p* < 0.001; Cohen's *d* = 0.747) but not for exemplars (*t*_(32)_ = −1.166; *p* = 0.252). Exemplars were associated with lower word frequency responses than features in both pre-cue (*t*_(32)_ = −7.275; *p* < 0.001; Cohen's *d* = 1.266) and retro-cue trials (*t*_(32)_ = −10.305; *p* < 0.001; Cohen's *d* = 1.794; Fig. 2*D*).

Intersubject variability was calculated as the number of different answers that were provided for each stimulus across all participants. A repeated measures ANOVA showed that variability was significantly higher for features compared with exemplars (main effect of task rule: *F*_(1,32)_ = 85.581; *p* < 0.001; partial *η*^2^ = 0.728). There was no significant effect of cue or interaction between the variables (both *p*s > 0.1; Fig. 2*E*).

### Responses with lower word frequency take more time

In order to explain variability in reaction times, we constructed five candidate mixed-effects regression models with different fixed-effect structures (Table 1, Behavioral GLMMs). Participants were added as random-effects predictors in all models. The best model (model 4) included word frequency and the interaction term between cue condition and task rule as fixed effects. The regression coefficients indicated a significant negative linear relationship of word frequency with reaction times (*β* = −0.021; *t* = −2.183; SE = 0.010; *p* = 0.030; Fig. 2*F*); that is, participants took more time to produce answers of low word frequency. There was also a significant main effect of cue (*β* = −0.196; *t* = −3.544; SE = 0.055; *p* < 0.001), and a significant interaction between cue and task rule (*β* = −0.088; *t* = −2.577; SE = 0.034; *p* = 0.010), similar to the results from the repeated measures ANOVA. The model had an overall fit of *R*^2^ = 0.206. In sum, word frequency predicted reaction times beyond the effects of cue and task rules.

### Pre-cue trials are associated with higher alpha activity than retro-cue trials

**Figure 3.**
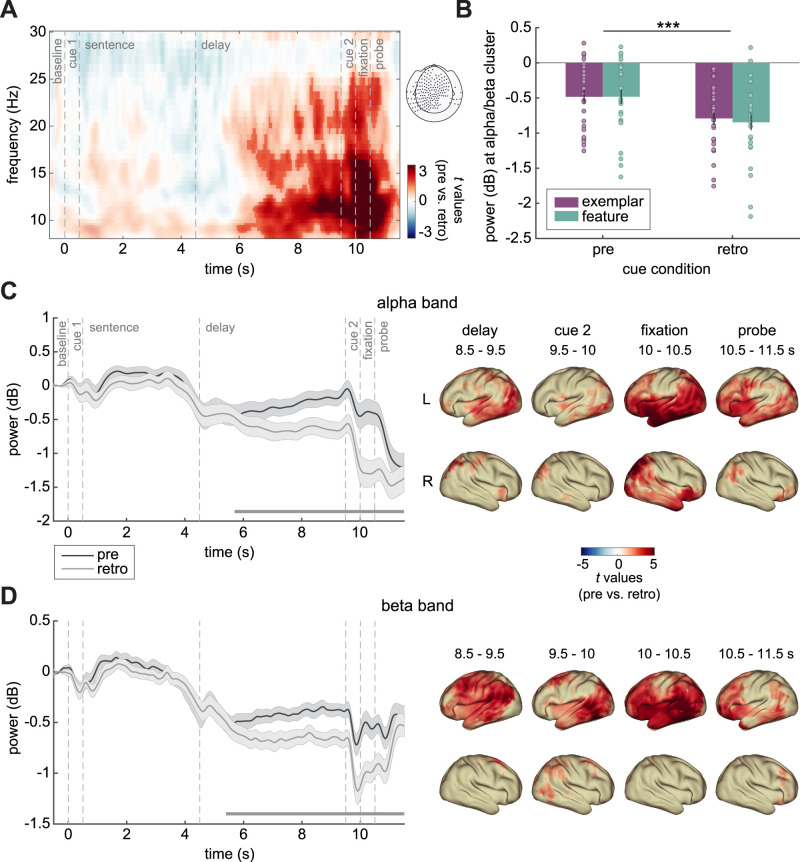
Time-frequency analysis comparing pre- versus retro-cue conditions. ***A***, TFRs of pre- versus retro-cue trials, showing *t* values averaged over significant sensors denoted in the topography (right), masked by significance (opacity mask). ***B***, Power averaged over significant sensors and time points (time window 4.9–11.5 s), separately for exemplar (purple) and feature (green) and for pre- and retro-cue conditions. Power values were converted to decibel by calculating the 10 times base 10 logarithm of the ratio versus baseline power (averaged from −0.45 to −0.25 s). ***C***, Left, Time courses of alpha power (8–13 Hz) for pre- (dark gray) and retro-cue conditions (light gray). Horizontal lines indicate significant time windows. Right, Source reconstructions for pre- versus retro-cue, showing *t* values exceeding the 95^th^ percentile. ***D***, Same as ***C*** for the beta band (14–25 Hz). Error bars represent ±1 SEM. ***p < 0.001.

We hypothesized pre-cue trials to be associated with higher alpha power during the delay period compared with retro-cue trials, due to lower cognitive load. A cluster-based permutation test contrasting pre- and retro-cue conditions indeed revealed a significant positive cluster (cluster-corrected *p* = 0.010) starting during the delay period (∼6 s) in the alpha band and extending to the beta band during the fixation and probe windows (after 10 s; Fig. 3*A*). This effect was not modulated by task rule, as a cluster-based permutation test showed no significant difference in time-frequency data for pre-cue minus retro-cue in exemplar versus feature (all *p*s > 0.3; Fig. 3*B*).

Source reconstruction revealed that effects were predominantly left lateralized in both the alpha (Fig. 3*C*) and beta bands (Fig. 3*D*). During the delay window, alpha effects peaked in lateral occipital cortex (LOC), inferior parietal lobule (IPL), as well as frontal regions (orbital gyrus, ORBG; paracentral lobule, PCL), while beta effects peaked in IPL and temporal regions (superior temporal gyrus, STG; fusiform gyrus, FG). During the second cue window, alpha effects involved mainly occipital (LOC) and motor regions (postcentral gyrus, PoCG; PCL), while beta effects were more temporally (posterior superior temporal sulcus, pSTS; inferior temporal gyrus, ITG; FG) and occipitally (LOC) localized. During the fixation window, alpha effects peaked in temporal (FG, STG, MTG, ITG) and frontal regions (ORBG, SFG), while beta effects peaked in temporal (ITG, FG, MTG, pSTS), frontal (ORBG), and parietal regions (IPL). Finally, during the probe, alpha effects were maximal in temporal (FG, STG, MTG) and frontal regions (ORBG, PrCG), while beta effects were mostly localized in frontal (MFG, IFG, ORBG) and to a lesser extent in temporal regions (FG, STG, ITG, MTG).

### Features are associated with higher beta activity than exemplars

**Figure 4.**
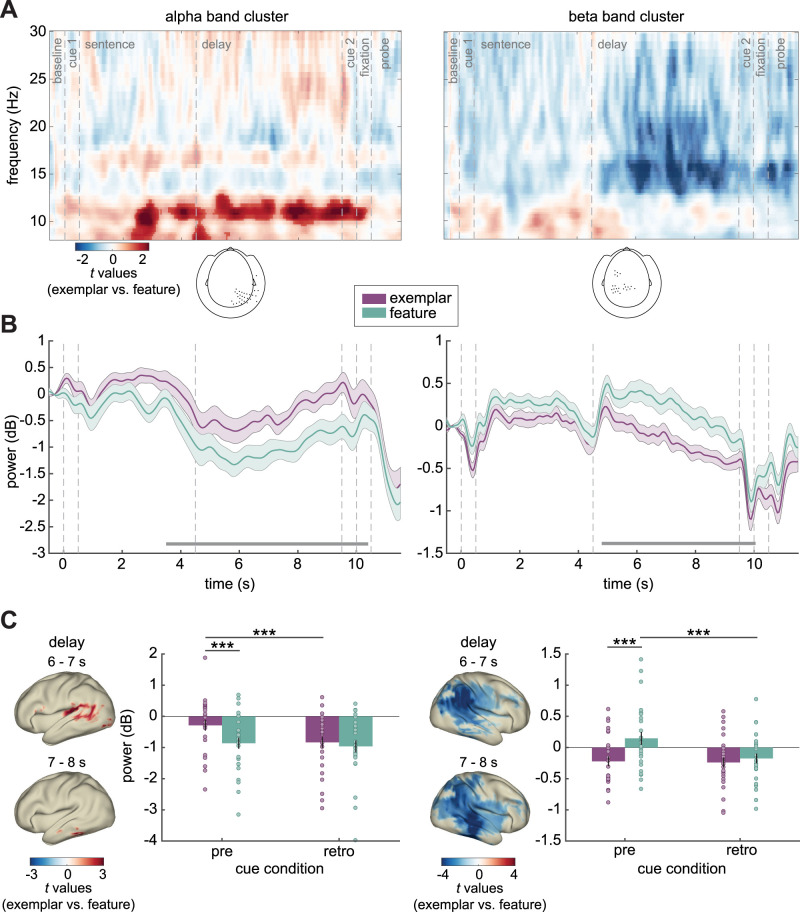
Time-frequency analysis comparing exemplar versus feature conditions. ***A***, TFRs of exemplar versus feature in pre-cue trials, showing *t* values averaged over significant sensors denoted in the topographies (bottom), separately for the alpha (left), and the beta band cluster (right), masked by significance. ***B***, Time courses of alpha (10–12 Hz) (left) and beta power (13–22 Hz) (right) for exemplar (purple) and feature (green). Horizontal lines indicate significant time windows. ***C***, Source reconstructions for exemplar versus feature in pre-cue trials, showing *t* values exceeding the 95th-percentile (left, alpha; right, beta). Bar plots show power averaged over significant sensors and time points (time window 6–8 s), separately for each condition. Error bars represent ±1 SEM. ***p < 0.001.

We hypothesized beta power to distinguish between different task rules, potentially linked to the recruitment of separate networks during response generation. We contrasted exemplar versus feature conditions using only pre-cue trials, as those contained information about the given task rule during the delay. In the alpha band, a cluster-based permutation test revealed a significant positive cluster (*p* = 0.014) starting halfway during stimulus presentation (∼2 s) and lasting until the probe. In the beta band, there was a significant negative cluster (*p* = 0.010) during the delay (Fig. 4*A*,*B*). Effects were indeed only present in the pre-cue but not in the retro-cue trials (confirmed by repeated measures ANOVAs in the identified significant cluster points; Fig. 4*C*).

Source reconstruction during the delay window revealed that alpha band effects were left hemispheric, initially peaking in the temporoparietal regions (STG, pSTS, IPL; 6–7 s) and then temporal regions (FG, ITG, MTG, STG; 7–8 s). In the beta band, effects were identified in the right hemisphere, peaking in temporoparietal regions (ITG, MTG, pSTS, FG, IPL).

### Reaction times linked to individual alpha peak frequency power

In order to investigate whether IAF power is linked to behavioral performance, we constructed mixed-effect regression models with reaction times as the dependent variable (Table 1, Neural GLMMs). Participants were added as random-effects predictors in all models. As there was no a priori hypothesis about the location of the effects, we constructed the models by averaging IAF power over sensors of each of seven regions of interest (ROIs), namely, central, left/right frontal, left/right temporal, left/right parieto-occipital cortex. The LRTs showed that all models were significantly better than the previous one except model 5, thus model 4 was picked as the best model. Furthermore, model 4 in the left frontal ROI was significantly better than that in all other ROIs.

**Figure 5.**
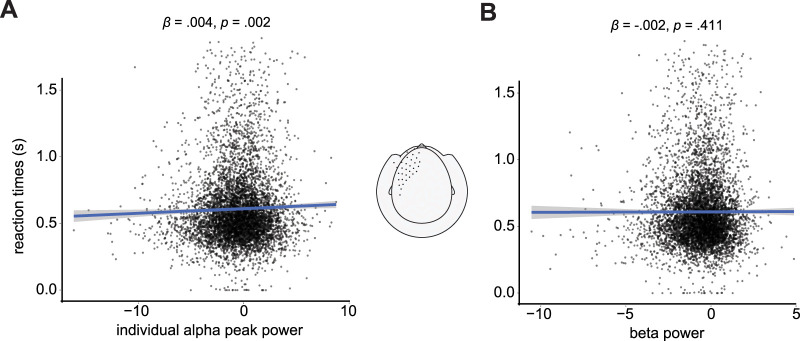
Relationship between neural measures and reaction times. ***A***, Scatterplot showing a positive relationship between IAF power in left frontal channels (denoted in the topography) and reaction times, as evidenced by a generalized linear mixed-effects model. ***B***, Same as ***A*** for the beta band.

The best model (model 4, left frontal) included IAF power and the main effects and interaction term between cue and task rule as fixed effects. Results showed a significant positive correlation between IAF power and reaction times (*β* = 0.004; *t* = 3.039; SE = 0.001; *p* = 0.002; Fig. 5*A*). There was also a significant effect of cue condition (*β* = 0.052; *t* = 2.698; SE = 0.019; *p* = 0.007), while the effect of task rule was not significant (*β* = −0.037; *t* = −1.884; SE = 0.019; *p* = 0.060). There was a significant interaction between cue and task rule (*β* = 0.054; *t* = 4.403; SE = 0.012; *p* < 0.001). The model had an overall fit of *R*^2^ = 0.217.

To test the frequency specificity of the effect, we conducted the same analysis for the beta band. Results revealed no significant effect of beta power on reaction times (*β* = −0.002; *t* = −0.823; SE = 0.002; *p* = 0.411; Fig. 5*B*). There was a significant effect of cue (*β* = 0.050; *t* = 2.601; SE = 0.019; *p* = 0.009), a marginal effect of task rule (*β* = −0.038; *t* = −1.958; SE = 0.019; *p* = 0.050), and a significant interaction between cue and task rule (*β* = 0.054; *t* = 4.436; SE = 0.012; *p* < 0.001). The model had an overall fit of *R*^2^ = 0.216.

## Discussion

Here, we constructed a novel rule-switching paradigm incorporating real-life functions from working memory and language comprehension to word production, in order to study the role of alpha and beta dynamics in a quasi-naturalistic setting. Participants were required to come up with an exemplar or a feature of a target word embedded in spoken sentences, as indicated by a pre- or retro-cue. As expected, reaction times of word production were slower for retro-cue compared with those for pre-cue conditions, likely due to higher cognitive load. Spectral results showed that alpha power during the delay window was lower for retro-cue compared with that for pre-cue in the left hemispheric language-related regions and that alpha power was negatively correlated with reaction times. These findings are in line with a role of alpha in facilitating task performance by regulating inhibition in regions linked to lexical retrieval. Importantly, the spatiotemporal pattern of beta activity was dissociated between exemplars and features in the right temporoparietal regions, but only when participants were aware of the task rule, which is in line with the proposed role of beta dynamics in the encoding of distinct categories and recruitment of their respective neural networks.

### Behavioral performance in the exemplar versus feature word production task

We observed that feature production was associated with longer reaction times compared with exemplar production. As this was a novel manipulation, we did not have an a priori prediction; however, a post hoc explanation might be found in the higher intersubject variability of responses for features compared with exemplars. Perhaps there were a large number of candidate feature responses for each target word to choose from, and thus the selection process took more time. On the contrary, the candidate exemplars were more limited, as evidenced by lower variability, and reaction times were shorter. These findings might also be linked to association strength, which refers to the percentage of people responding with a certain word when presented with the same target word (Hutchison, 2003). Indeed, the strength of the connection between words affects the rate of spread of conceptual activation and, consequently, the speed of retrieval. Reaction times in a verb generation task are shorter when words have high association strength, and longer when there is no high association strength (Martin and Yan, 2006). Association strength is also correlated with the degree of semantic relation between words (Prior and Geffet, 2019), which might further explain why features, which showed low association strength, took longer to produce compared with exemplars. Furthermore, word frequency was lower for exemplars than for features. We suspect that there is a baseline difference between word frequency and association strength of exemplars versus features, but this remains to be investigated in future studies. Finally, reaction times were associated with word frequency, with faster retrieval of common words. This effect was over and above any differences in reaction times for exemplars versus features. It is an expected finding given that lexical selection in speech production is sensitive to word frequency, such that more uncommon words are associated with slower retrieval (Navarrete et al., 2006).

### Alpha power is linked to inhibition and behavioral performance

As expected, alpha power was modulated by the informativeness of the cue; that is, we observed lower alpha power during the delay for retro-cue compared with pre-cue trials. In the retro-cue condition, participants did not yet know the task rule and had to come up with both an exemplar and a feature, which meant higher cognitive demands than in the pre-cue condition, in which they only needed to come up with one word. The alpha power modulation was most pronounced in left hemispheric occipital, parietal, and frontal areas and extended to the beta band in a range of parietal and temporal areas. A previous retro-cueing study found decreased prefrontal and occipitoparietal alpha and beta power with increased cognitive load (ElShafei et al., 2022). Indeed, beta oscillations have also been linked to functional inhibition, potentially by activating inhibitory neurons or saturating excitatory neurons (Ede et al., 2011; Spitzer and Haegens, 2017; Miller et al., 2018). Notably, the direction of the effect seems to be largely dependent on the task. Studies finding posterior alpha decreases during memory maintenance argue that this supports engagement of task-relevant areas to support storage of information (van Ede et al., 2017; de Vries et al., 2020). On the contrary, studies showing increased alpha power during memory maintenance suggest that this serves to block potential distractors by inhibiting occipital/parietal task-irrelevant areas (Jensen et al., 2002; Tuladhar et al., 2007; Bonnefond and Jensen, 2013). Indeed, there is evidence suggesting that there might be different sources of alpha oscillations in the brain reflecting the same underlying mechanism (Klimesch, 1999; Rodriguez-Larios et al., 2022). In our study, participants need to keep the task rule in memory throughout the whole delay window; therefore, reduced alpha power in task-relevant regions is reduced to support storage of information.

Previous work also demonstrated that alpha and beta dynamics subserve language comprehension by contributing to high-level operations, such as processing of syntactic dependencies (Meyer et al., 2013; Zioga et al., 2023) and predictive processing in structured sentences (Bastiaansen et al., 2010; Lewis and Bastiaansen, 2015). Effects in beta might also reflect increased demands for memory retrieval of latent language-specific knowledge representations (Hagoort, 2013). The left temporal regions have been shown to play a role in lexical retrieval (Ouden et al., 2012; Klaus et al., 2019), as well as in linking phonological to semantic networks (Matchin and Hickok, 2020).

Importantly, we found that individual alpha power in left frontal areas negatively correlated with reaction times. Previous studies reported associations between alpha dynamics and behavior (Luft et al., 2018; ElShafei et al., 2022), with alpha power in task-relevant regions negatively correlated with behavioral performance (Haegens et al., 2011a, 2012; van Ede et al., 2017; ElShafei et al., 2022). Critically, activation of left frontal regions has been associated with word generation requiring selection among competing alternatives (Martin and Yan, 2006). We speculate that oscillatory dynamics play a role in facilitating task performance by regulating inhibition in task-relevant areas. Our task required participants to perform a series of processes, including the generation of exemplars and features, selection of one of the competing concepts, maintenance in working memory, and verbalization. The observed effects in left temporal regions might reflect release from inhibition of task-relevant regions linked to memory retrieval and lexical processes during conceptual generation and selection. Furthermore, intracranial research showed that the left frontal regions encode anticipation of composition in broadband low frequencies and exhibit substantial connectivity with pSTS (Murphy et al., 2022). Overall, our findings suggest that both alpha and beta supported the enhanced engagement of task-relevant areas during higher load; however, only alpha correlated with behavioral performance.

We also found lower alpha power for feature than exemplar production, an effect spatially constrained to the left temporoparietal regions, peaking in STG, pSTS, and IPL. This might be due to higher cognitive demands for feature production, in line with our behavioral findings. fMRI studies found STS involvement in semantic tasks requiring semantic categorization or judgements of semantic properties of words (Gitelman et al., 2005; Gold et al., 2006), and it has been suggested that the left pSTS is involved in long-term storage of lexical-semantic representations (Lau et al., 2008; Gow, 2012). Furthermore, a large body of research has demonstrated the role of temporoparietal regions in both word- and sentence-level processing (Friederici and von Cramon, 2000; Meyer et al., 2002; Dronkers et al., 2004; Rodd et al., 2005; Berwick et al., 2013; Zaccarella et al., 2017). In our study, we did not use free speaking or conversation, as we wanted to control cognitive load and test potential interactions with task rule. Nevertheless, our paradigm requires fewer steps of inference between experimental findings and behavior compared with prior studies. Future research is needed to test the observed effects in a more naturalistic speech setting.

### Beta power dissociates distinct task rules

We observed a distinct spatiotemporal pattern of oscillatory dynamics comparing exemplar and feature production. Specifically, the feature condition showed higher beta power during the delay in a range of right-hemispheric regions, especially temporoparietal cortex. Importantly, this was only the case for pre-cue trials when participants knew the task rule, whereas the effect was absent in retro-cue trials when the task rule was not yet known. According to a proposal by Spitzer and Haegens (2017), beta oscillations support the (re)activation of latent content representations by supporting the transition of a latent item to an active working memory state (Antzoulatos and Miller, 2016; Rose et al., 2016). This proposal is based on a range of studies showing content-specific beta modulations in working memory tasks when participants recall previously encoded information (Spitzer and Blankenburg, 2011; Spitzer et al., 2014; Wimmer et al., 2016), task rules (Buschman et al., 2012), and decision outcomes (Haegens et al., 2011b, 2017). In a recent nonhuman primate study using a categorization task, beta activity supported the reactivation of cortical representations by mediating neural ensemble formation within and between brain regions (Rassi et al., 2023). Crucially, two distinct beta rhythms were consistently associated with the same two categorical outcomes even when categorical boundaries shifted and reflected the animal's decision including in case of errors. Another study found evidence for category-specific patterns of beta synchrony in the PFC, especially for stimuli near category boundaries, interpreted as a top-down mechanism of gain control (Stanley et al., 2018). Further, beta has been suggested to play a role in top-down, executive control of working memory storage (Miller et al., 2018). We speculate that in our current study, beta modulations reflect the given task rule held in mind during the delay via the recruitment of task-related neural networks. We thus hypothesize that the exemplar versus feature differences in beta activity are due to recruitment of distinct networks for each task rule. However, future studies are needed to test whether beta power is modulated by the number of rule representations. Further research is also needed to elucidate whether task rules are encoded parametrically as a function of linguistic demands. As discussed in the section above, “Alpha power is linked to inhibition and behavioral performance,” the effect of lower left temporoparietal alpha power for feature than exemplar might be attributed to higher cognitive demands for feature production, in line with our behavioral findings. Therefore, here alpha and beta seem to serve two different functions linked to cognitive load and task rule encoding, respectively.

Overall, our work fills a gap in the existing research by investigating the alpha and beta oscillatory dynamics associated with language comprehension and production. It comprises the first observation of alpha and beta modulations based on cognitive load and task rule in a linguistically demanding task. Neurolinguistics has been primarily focused on low-frequency dynamics during language comprehension and less in production. For instance, it is thought that low-frequency oscillations rhythmically modulate neuronal excitability, enhancing speech processing by temporally aligning with the landmarks of the acoustic stream (Giraud and Poeppel, 2012; Doelling et al., 2014). Here, we studied exemplar versus feature selection, which relies on complex knowledge, not only of semantic memory, but also of linguistic (lexical) aspects. The fact that we observed effects of word frequency on reaction times and that word frequency was modulated by task rule suggests that the thinking period involved processes related to lexical knowledge. It is not trivial to disentangle linguistic from domain-general processes that take place concurrently. However, as here the contents of working memory are lexical, the selection process taps into linguistic information, which is corroborated by the fact that reaction times were modulated by a lexical feature, that is, word frequency. Finally, the selection of a word largely depends on task demands, focusing on what is relevant and inhibiting distracting information. It is those domain-general processes that subserve the generation and selection of task-relevant linguistic concepts that we believe are reflected in the observed alpha and beta modulations in our task.

### Conclusion

We constructed a novel rule-switching paradigm incorporating linguistic demands. Overall, our results show that alpha power is modulated by cognitive load and is linked to task performance, potentially by regulating inhibition in brain regions linked to lexical retrieval. Additionally, the spatiotemporal pattern of beta activity differed between two distinct task rules in right temporoparietal regions, in line with the proposed role of beta in encoding of distinct categories and recruitment of respective neural networks. Overall, our study provides evidence for the generalizability of the role of alpha and beta oscillations from low-level sensory to complex linguistic processing and supports the view of a domain-general functional role of cortical oscillations across the hierarchy of cognitive processes.

## Synthesis

Reviewing Editor: Ifat Levy, Yale School of Medicine Decisions are customarily a result of the Reviewing Editor and the peer reviewers coming together and discussing their recommendations until a consensus is reached. When revisions are invited, a fact-based synthesis statement explaining their decision and outlining what is needed to prepare a revision will be listed below. The following reviewer(s) agreed to reveal their identity: Elliot Murphy, Polina Iamshchinina.

This MEG study examined rule-switching during word production. Participants listened to sentences and had to produce either a word of the same category as the last noun in the sentence, or a feature of that noun. The cue indicating which rule is valid in each trial could be shown before or after the sentence (pro- vs retro-cue manipulation). The authors document lower alpha power for retro-cue compared to pre-cue trials in left language regions. Alpha power also negatively correlated with reaction times. Within the two trial types, the authors also showed different spatiotemporal patterns of beta activity in right temporo-parietal regions.

These results are consistent with the authors' motivation to make a cognitively realistic connection between lower-level perceptual processes and the existing literature on language comprehension in low frequency spaces.

The motivation for the paper is clear, the approach is clearly explained, and the results are largely convincing, and can make an important contribution to the literature on the general role of alpha and beta oscillations. Reviewers noted, however, several issues that must be addressed, and made some suggestions, as detailed below.

- The motivation for the specific bands (alpha, beta) is clear, but there are no anatomical constraints on the analysis or hypothesis. The authors predict "a differentiated spatiotemporal profile of beta-band activity" for exemplar vs. feature trials, but since they are using MEG, perhaps they could be more specific as to in which regions they might expect to see this? The analysis has of course already been conducted, and the results are quite clear, but some more general direction to what we could have expected based on current literature would be helpful. For example, some work has tried to chart out language from non-linguistic regions (Fedorenko & Varley 2016), and a recent paradigm explored the interactions of lexical and structural information during sentence composition to carve out the frontotemporal language network (Woolnough et al. 2023). These frameworks and findings should be used to clarify what kind of contribution this manuscript aims to make. Likewise, when the authors discuss left-hemisphere language-related regions, they should ideally be more explicit here, especially given that we might expect the non-linguistic lower-level engagement to presumably tax extra-linguistic regions. For example, the effects in Fig. 4C are interestingly covering posterior temporal language sites but other sites too.

- Even though the authors report the results in accordance with their hypotheses, the results figures show that both the load effect and the task rule difference are present both in alpha and in beta bands. Hence, the questions: How specific is the load effect to the alpha band in this study? The task rule difference is reflected not only in beta-band power differences but also in alpha band and the modulation in alpha band is opposite to the beta band. The alpha-band effect is not described in the discussion section. How specific is the task rule difference effect to the beta band?

- A feature of a given exemplar (e.g., shrimp) could be unique to the exemplar (e.g., has many legs) or shared across the entire category (e.g., pink as well as tuna), thus leading to shared representations of the exemplar- and feature- rules. Did the authors control for such possible overlaps?

- In addition to pure reaction time differences between exemplar and feature trials, is it possible that there are semantic search differences, based on the large number of candidate feature responses relative to the small selection of exemplar answers?

- Several studies show that posterior alpha power increases with memory load (e.g., Hu et al., 2019; Jensen et al., 2002) as well as alpha in frontocentral regions (e.g., Manza, Hau & Leung, 2014), whereas in the current study, alpha-power decreases with load. It would be interesting to learn the authors' interpretation of the difference between their and previous results.

- Predicting reaction times from individual alpha peak frequency (IAF) power: why exactly 20 sensors with the highest power? Within what time period in the trial was this frequency bin (8-13 Hz) identified? Why highest power is the best criterion for correlation with behavior?

- Lines 419-420 (and later on lines 510-512): "the spatiotemporal pattern of beta activity was dissociated between exemplars and features in right temporo-parietal regions, but only when participants were aware of the task rule..." - since the instruction to participants was to both think about a feature and an exemplar in the retro-cue task, it is possible that the inability to differentiate the two rules in the retro-cue task was due to an overlap of the rule representations. Thus, the difference in the pro-cue task may be due to single representations of rules, rather than rule awareness.

- While the authors do a nice job of elucidating their alpha/beta dynamics in RH regions, the manuscript does not seem to clearly tie these results back to neurolinguistics. How do these signals complement our understanding of LH language low frequency dynamics? The authors could, For example, frame this story better if they discuss examples in the literature where opposing perspectives have been aired (i.e. oscillations either are, or are not, domain-general signatures etc).

- The authors may consider running a supplementary analysis of theta power dynamics, given that theta has more commonly been implicated in the language literature for semantic and lexical processing (Pu et al. 2020). Beta dynamics, as the authors note, have been related to content-specific dynamics, but it's also potentially interesting that the difference between exemplar vs. feature is one of categorial processing (within-item quality vs. higher-order categorization). The fact that the authors found different reaction times for features vs. exemplars further helps motivate this exploratory goal. This is left for authors' discretion.

- Minor comments:

- The first two sentences of the abstract seem a bit too conceptual (what are the basic perceptual functions and more complex processes implied?), making them a bit more specific could help pose the problem earlier.

- The term "inverse word frequency" may be challenging for the manuscript comprehension. The authors should consider using "word frequency", or alternatively add interpretations to the inverse word frequency when they mention it.

- It would be helpful if the authors could add the names of the events and motivation for the choice of the time and frequency windows to the source reconstruction description in the Methods.

- The authors may want to consult intracranial research that supports a left frontal role for phrasal prediction, localizing low frequency signatures (Murphy et al. 2022). Likewise, in the discussion of beta power some recent frameworks have tried to develop these themes (Miller et al. 2018). The report in Stanley et al. (2018) is relevant here.

References

Fedorenko, E., & Varley, R. (2016). Language and thought are not the same thing: evidence from neuroimaging and neurological patients. Annals of the New York Academy of Sciences 1369(1): 132-153.

Miller, E.K., Lundqvist, M., & Bastos, A.M. (2018). Working memory 2.0. Neuron 100(2): 463-475.

Murphy, E., Woolnough, O., Rollo, P.S., Roccaforte, Z.J., Segaert, K., Hagoort, P., & Tandon, N. (2022). Minimal phrase composition revealed by intracranial recordings. Journal of Neuroscience 42(15): 3216-3227.

Pu, Y., Cheyne, D., Sun, Y., & Johnson, B.W. (2020). Theta oscillations support the interface between language and memory. NeuroImage 215: 116782.

Stanley, D.A., Roy, J.E., Aoi, M.C., Kopell, N.J., & Miller, E.K. (2018). Low-beta oscillations turn up the gain during category judgments. Cerebral Cortex 28(1): 116-130.

Woolnough, O., Donos, C., Murphy, E., Rollo, P.S., Roccaforte, Z.J., Dehaene, S., & Tandon, N. (2023). Spatiotemporally distributed frontotemporal networks for sentence reading. Proceedings of the National Academy of Sciences 120(17): e2300252120.
